# A Critical Review of the Drug Promotional Literature Published in Scientific Medical Journals and Available at Outpatient Departments: A Cross-Sectional Observational Study

**DOI:** 10.7759/cureus.31283

**Published:** 2022-11-09

**Authors:** Kavita Vivek, Pradnya Deolekar, Azra Naseem, Deepakkumar G Langade, Pramila Yadav

**Affiliations:** 1 Pharmacology, D.Y. Patil Deemed to be University, Navi Mumbai, IND

**Keywords:** drug promotional literature, pharmaceutical marketing, marketing, advertisement, pharmaceutical company, drug promotion

## Abstract

Background

A vital method used by pharmaceutical companies to make physicians aware of new drugs and increase the prescription and sale of the same is through drug promotion literature (DPL) published in scientific journals and distributed in outpatient departments (OPDs). It is important that drug promotion is done ethically to avoid the spread of false information for which guidelines are available at the international level by the World Health Organization (WHO) and at the national level by the Organisation of Pharmaceutical Producers of India (OPPI). In this study, we aim to review the DPLs used for promotion by market authorization holders (pharmaceutical business entities) in scientific healthcare journals and OPDs for their compliance with the “Ethical criteria for medicinal drug promotion” of the WHO and OPPI Code of Ethics Practice. In addition, we compare the ethical standard of the DPL available in scientific journals and OPDs with respect to existing norms and guidelines.

Methodology

A cross-sectional, observational study was conducted at a tertiary care teaching institute in Navi Mumbai, India. DPLs were collected from journals available at the institute library nearby published from January-June 2022 and from the outpatient departments of our hospital and other clinics nearby during the same time duration. Analysis was done according to the criteria given in WHO and OPPI guidelines. Each point in the criteria was scored as 1 or 0 based on whether the DPL was compliant or not respectively. DPLs were graded into 3 categories based on percentage compliance: Grade A (>70%), Grade B (35-70%), and Grade C (<35%).

Results

A total of 370 DPLs were collected, of which 191 (51.6%) were collected from scientific journals and 179 (48.4%) from OPDs. DPLs collected from journals showed that only 7.85% belonged to Grade A (WHO guidelines). According to the OPPI guidelines, 57.59% of the same DPLs belonged to Grade A. DPLs from OPDs showed similar results by both guidelines with >90% belonging to Grade B. Approximately less than 5% of the DPLs belonged to Grade C from both scientific journals and OPDs.

Conclusions

None of the DPLs were found to be entirely compliant with either of the guidelines. Most of the DPLs from both sources belong to Grade B, with information about adjuvants, adverse drug reactions, contraindications, drug interaction, and references to scientific literature missing from them. DPLs belonging to Grade C even had information about active ingredients missing from them which can lead to serious harm due to the wrong prescription of drugs.

## Introduction

The pharmaceutical industry is growing faster than ever with new drugs coming up every single day and continuous improvements taking place in the treatment modalities. A steady rate of growth has been reported over the last two decades by Food and Drug Administration when it comes to approving novel drugs [1]. The onus to make physicians aware of new drugs and their beneficial effects lies on the marketing authorization holders. The important role of drug marketing comes into the picture here.

A vital method used for this purpose is drug promotion literature (DPL) published in scientific journals and distributed to physicians through medical representatives in outpatient clinics [2]. The main aim of this is to successfully market and increase the subsequent sale of a particular brand of the drug. To ensure that monetary gain for companies does not take over the essence of improving the healthcare standards of the country, it is important to ensure that drug promotion is done ethically. The reliance of treating physicians on information provided in these DPLs is extensive which further adds to the need for it to fulfill all ethical standards [3]. Many medical practitioners have an extremely busy schedule because of the workload, especially in a country like India. Hence, they find less time to enhance their knowledge through continuing medical education (CME) lectures and rely on DPLs to get information about new developments in therapeutics [4].

Internationally, ethical guidelines for drug promotional activities have been defined by the Ethical criteria for medicinal drug promotion of the World Health Organization (WHO), 1988 [5], and the Code of Pharmaceutical Marketing Practices of the International Federation of Pharmaceutical Manufacturers & Associations (IFPMA) [6]. Nationally, drug promotion is governed by the guidelines provided by the Organisation of Pharmaceutical Producers of India (OPPI) [7] and national legislation.

This study aimed to review the DPLs collected from scientific healthcare journals and outpatient departments (OPDs) according to the WHO and OPPI criteria and compare the ethical standard of the DPLs with respect to existing norms and guidelines. This study finds its due importance in the fact that incomplete information in this literature can lead to irrational prescriptions of drugs that can have a negative impact on the healthcare service practitioners provide [8]. Studies like these if they find that the ethical guidelines are not being followed can pave the way for more strict regulations to be introduced by authorities.

This article was previously presented as a poster at the National Conference of Andhra Pradesh Pharmacologists Society (APPSCON) on September 24, 2022.

## Materials and methods

A cross-sectional, observational study was conducted at a tertiary care teaching institute in Navi Mumbai, India. Study approval was received from the Institutional Ethics Committee on August 11, 2022, (reference number: 2022/090). DPLs were collected from scientific journals available at the institute library published from January to June 2022. We collected DPLs from OPDs of our hospital and other clinics nearby from January to June 2022. The convenience sampling method was used. DPLs for medical devices, prostheses, and Ayurvedic medicine were excluded from our study. DPLs repeated in different issues of the same journal or in a different journal were also excluded. DPLs were reviewed according to the criteria provided by the WHO [5] and OPPI [7].

WHO criteria for ethical promotion of drugs

(1) The company should mention the name(s) of the active ingredient(s) using either the international non-proprietary names or the approved generic name of the drug. (2) The brand name should be provided. (3) The content of the active ingredient(s) per dosage form or regimen should be mentioned. (4) Mention should be made of the names of ingredients known to cause problems. (5) The advertisement should mention the approved therapeutic uses of the drug. (6) Details of the dosage form or regimen must be provided. (7) The side effects or major adverse drug reactions should be mentioned. (8) The precautions, contraindications, and warnings should be listed. (9) The advertisement should mention major interactions with other drugs. (10) The name and address of the manufacturer or distributor should be provided. (11) Reference should be made to scientific literature, as appropriate.

OPPI code of ethical practice

1. The name of the product (brand name). (2) The active ingredients. (3) The name and address of the pharmaceutical company or its marketing agent. (4) The date of production of the advertisement. (5) Approved indications. (6) Dosage. (7) Method of use. (8) Succinct statement of contraindications, precautions, and side effects.

Each point in the criteria was scored as 1 or 0 based on whether the DPL was compliant or not, respectively. Scoring of DPL according to the WHO criteria was done out of 11, and according to the OPPI criteria was done out of 8. DPLs were graded into three categories based on percentage compliance: Grade A (>70%), Grade B (35-70%), and Grade C (<35%).

Statistical analysis

All data were collected in Microsoft Excel (version 16.62). Descriptive statistics were applied. Data were analyzed as proportions and percentages. Wherever necessary, the results are depicted in the form of tables and graphs.

## Results

**Figure 1:**
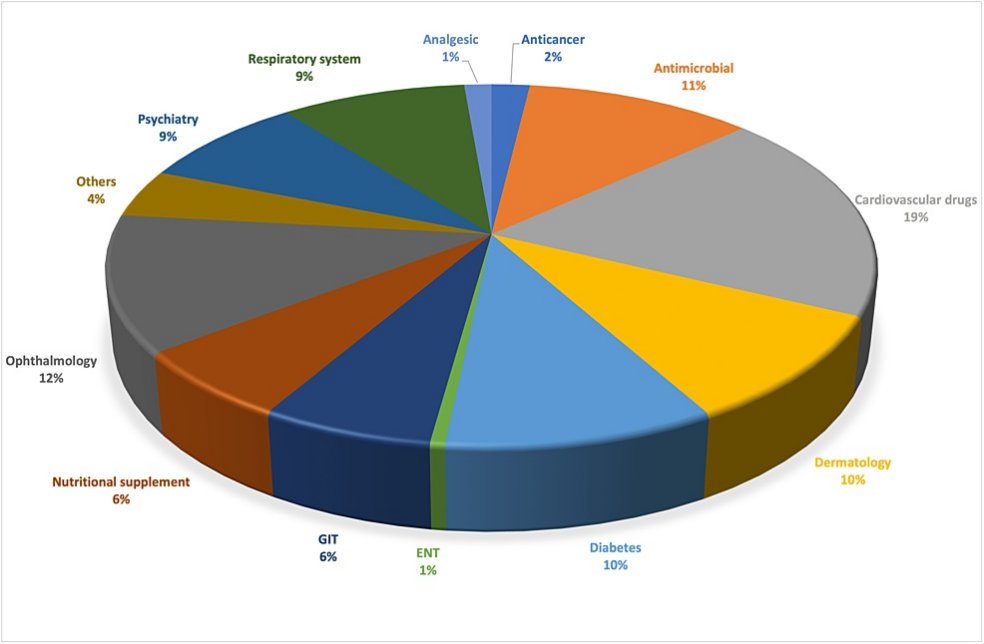
Classification of drug promotional literature according to the type of drug promoted (n = 370). GIT: gastrointestinal, ENT: ear, nose, and throat

A total of 62 journals published from January to June 2022 were analyzed. From these journals, we got a total of 191 DPLs. A total of 179 DPLs were collected from OPDs. Figure 1 shows a graphical representation of the classification according to the type of drug promoted.

Analyses of DPLs collected from scientific journals and OPDs according to the WHO and OPPI criteria are presented in Table 1 and Table 2, respectively.

**Table 1:** Analysis of drug promotional literature according to the WHO criteria. DPL: drug promotional literature; WHO: World Health Organization; OPD: outpatient department

WHO criteria	DPLs from scientific journals		DPLs from OPD	
	Number	Percentage (%)	Number	Percentage (%)
Name of the active ingredient	186	97.38	169	96.41
Brand name	191	100.00	179	100.00
Content of the active ingredient per dosage form	170	89.01	165	92.18
Name of the other ingredient known to cause problems	0	0.00	0	0.00
Approved therapeutic uses of the drug	111	58.12	121	67.60
Dosage form or regimen to be followed	186	97.38	149	83.24
Side effects or major adverse drug reactions	15	7.85	2	1.12
Precautions, contraindications, and warnings	15	7.85	2	1.12
Major interaction with other drugs	8	4.19	2	1.12
Name/address of the manufacturer/distributor	190	99.48	179	100.00
Reference to the scientific literature	34	17.80	15	8.38

**Table 2:** Analysis of drug promotional literature according to the OPPI criteria. DPL: drug promotional literature; OPPI: Organisation of Pharmaceutical Producers of India; OPD: outpatient department

OPPI criteria	DPLs from scientific journals		DPLs from OPD	
	Number	Percentage (%)	Number	Percentage (%)
Name of the active ingredient	186	97.38	169	94.41
Brand name	191	100.00	179	100.00
Name/address of the manufacturer/distributor	190	99.48	179	100.00
Date of production of advertisement	191	100.00	0	0.00
Approved indications of the drug	111	58.12	121	67.60
Dosage	2	1.05	2	1.12
Method of use	186	97.38	149	83.24
Precautions, side effects, and contraindications	15	7.85	2	1.12

**Figure 2:**
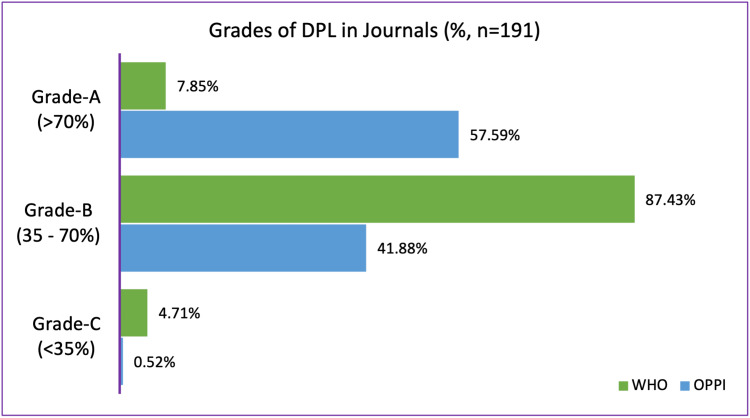
Comparison between drug promotional literature collected from scientific journals according to the WHO and OPPI criteria. DPL: drug promotional literature; WHO: World Health Organization: OPPI: Organisation of Pharmaceutical Producers of India

Figure 2 shows a graphical representation of the comparison between DPLs collected from journals according to the WHO and OPPI criteria.

**Figure 3:**
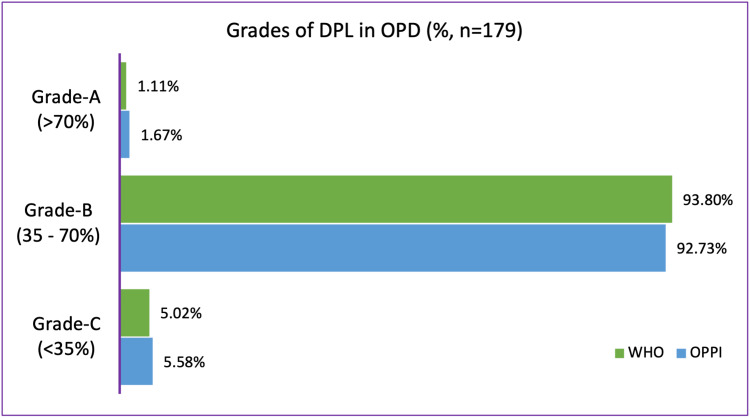
Comparison between drug promotional literature collected from outpatient departments according to the WHO and OPPI criteria. DPL: drug promotional literature; WHO: World Health Organization: OPPI: Organisation of Pharmaceutical Producers of India

Figure 3 shows a graphical representation of the comparison between DPLs collected from OPDs according to the WHO and OPPI criteria.

**Figure 4:**
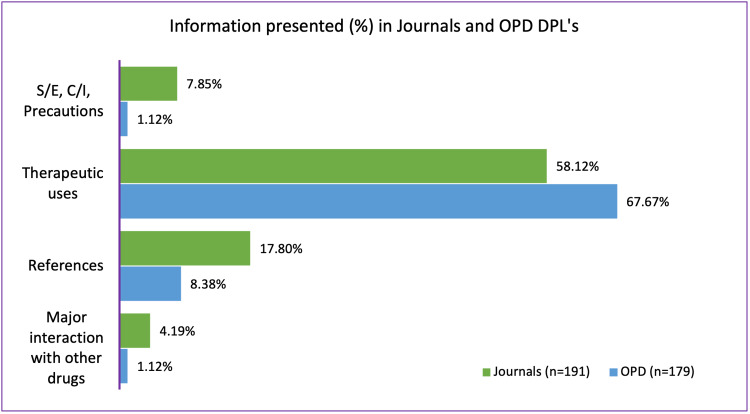
Difference in the information presented in the drug promotional literature from scientific journals and outpatient departments. DPL: drug promotional literature; OPDs: outpatient departments; S/E: side effects; C/I: contraindications

Figure 4 shows the important differences in the information presented in DPLs in journals and OPDs.

## Discussion

DPLs have been used since time immemorial to spread relevant information about new drugs coming into the market and increase the prescription and sale of the drugs.

In 1930, Sri Ram Nath Chopra constituted a Drug Inquiry Committee in India which analyzed drug pamphlets and found out that spurious claims were being made about drugs being sold in the market. This happened much before the WHO took care of this threat in 1988 [9]. When due attention was given to ensuring that drug promotion is done ethically, certain guidelines were laid down at the international and national levels. Over time, several studies have been conducted to check the compliance of DLP to the criteria mentioned in the guidelines, but the results have been nothing but disappointing. Most studies have found that most of the DPLs analyzed are not entirely compliant with the guidelines [10-13].

Out of the 191 DPLs collected from scientific journals and 179 collected from the OPDs, none were found to be compliant with any guidelines completely. Because we collected DPLs across multiple specialties, observations made in this study can be extended to most branches of medicine.

The grading of DPLs collected from journals according to the WHO guidelines showed that only 7.85% belonged to Grade A. Most DPLs (87.43%) belonged to Grade B. The key information missing from most of these DPLs in Grade B were details of other ingredients known to cause problems, adverse effects of the drug, precautions, contraindications, warnings, interactions with other drugs, and reference to scientific literature. Such details are relevant to the safe and adequate use of new drugs coming into the market, but the information appears to be missing from most published DPLs. The guidelines given by OPPI are less stringent with information about adjuvants, interaction with other drugs, and reference to scientific literature not mandatory. Hence, the same DPLs collected from journals when analyzed according to the OPPI guidelines showed a better result, with 57.59% belonging to Grade A.

The grading of the DPLs collected from OPDs showed a very similar result by both the WHO and OPPI guidelines. More than 90% of the DPLs belonged to Grade B with relevant information missing from most of them. Around 5% of the DPLs belong to Grade C. An astonishing finding was the fact that DPLs belonging to Grade C just included information about the brand name, therapeutic use of the drug or the dosage form, and the name/address of the manufacturer. Details as basic as the active ingredient present in these drugs were not included. This can have major implications as the wrong drug can be prescribed to patients. The overall purpose of publishing and distributing DPLs is lost when the guidelines are not followed. Previous studies conducted by Dhodi et al. [14], Priyanka et al. [15], and Jadhav et al. [16] showed that all DPLs analyzed had the active ingredient, brand name, and therapeutic use of the drug but we did not find this in our study, especially in the DPLs collected from the OPD.

The DPL published in journals had more information about precautions, side effects, contraindications, interaction with other drugs, and references to scientific literature. This information is rarely found in DPLs published in the OPDs. We found this finding to be similar to a previous study conducted by Puttaswamy et al. [17]. Therapeutic uses of the drug were found to be more in the DPLs in OPD (58.12%) compared to scientific journals (67.67%) but the difference is not large.

The findings of this study have been disappointing, to say the least. The lack of crucial information can have serious implications on the prescription and sale of drugs which might give monetary benefit to the pharmaceutical industry but would be extremely harmful to the health of patients. Analysis of more DPLs would give us better clarity about the overall compliance by marketing authorization holders to these guidelines.

## Conclusions

None of the DPLs were found to be fulfilling any guidelines in their entirety. Most DPLs had information about side effects, contraindications, precautions, interactions with other drugs, references to scientific literature, and information about other ingredients known to cause problems missing from them. Stricter regulations need to be introduced by the government to ensure that the situation improves. It is also essential for physicians to gradually develop awareness about these guidelines so that they can critically analyze them before they trust the information provided in the DPLs.
